# A Novel Density of States (DOS) for Disordered Organic Semiconductors

**DOI:** 10.3390/mi14071361

**Published:** 2023-06-30

**Authors:** Dong Qin, Jiezhi Chen, Nianduan Lu

**Affiliations:** 1 School of Information Science and Engineering, Shandong University, Qingdao 266237, China; dongqin@mail.sdu.edu.cn (D.Q.); chen.jiezhi@sdu.edu.cn (J.C.); 2 State Key Lab of Fabrication Technologies for Integrated Circuits & Laboratory of Microelectronics Devices and Integrated Technology, Institute of Microelectronics, Chinese Academy of Sciences, Beijing 100029, China

**Keywords:** disordered organic semiconductors, density of states, mobility, concentration, electric field

## Abstract

In this work, we proposed a novel theory of DOS for disordered organic semiconductors based on the frontier orbital theory and probability statistics. The proposed DOS has been verified by comparing with other DOS alternatives and experimental data, and the mobility calculated by the proposed DOS is closer to experimental data than traditional DOS. Moreover, we also provide a detailed method to choose the DOS parameter for better use of the proposed DOS. This paper also contains a prediction for the DOS parameters, and it has been verified by the experimental data. More importantly, the physical meaning of the proposed DOS parameter has been explained by equilibrium energy theory and transport energy theory to make this proposed model more rational. Compared with the improved DOS based on Gaussian and exponential DOS, this work is a new attempt to combine probabilistic theory with physical theory related to DOS in disordered organic semiconductors, showing great significance for the further investigation of the properties of DOS.

## 1. Introduction

Over the past decades, charge carrier transport in organic semiconductors has been extensively studied because of its importance to organic semiconductor device performances [1,2]. It is well known that the charge carrier transport in most organic semiconductors is performed in a disordered system, unlike inorganic semiconductors [3,4]. Thus, it is difficult to create a unified model to explain the carrier transport of disordered organic semiconductors under different conditions.

Physically, the density of states (DOS), determining the electronic and optoelectronic properties, is very important for carrier transport [5]. Up to now, there was no direct experimental evidence verifying specific DOS models. Thus, the most common method of determining the DOS is to compare experimental data with the appropriate theory using some trial DOS functions between experimental and theoretical results [6]. Currently, the most common DOS models used are the Gaussian DOS model ($g\left(E\right)=\frac{N_{t}}{\sqrt{2\pi }\sigma_{G}}\exp\left(-\frac{{\left(E-b_{G}\right)}^{2}}{2\sigma_{G}^{2}}\right)$) and the exponential DOS model ($g\left(E\right)=\frac{N_{t}}{\sigma_{E}}\exp\left(-\frac{E-b_{E}}{\sigma_{E}}\right)$) [7,8,9,10,11,12,13]. Although the two DOS models work well under different situations, their limitations are also evident. For example, when calculating mobility, the exponential DOS does not work at low concentrations, and the Gaussian DOS does not work at high concentrations [6,14]. More importantly, as stated by some researchers, the Gaussian DOS model is only an assumption and may not strictly be true, and the DOS of disordered organic semiconductors cannot be purely exponential at low concentration [6,7]. Therefore, modification of Gaussian DOS and exponential DOS is a common method to deal with the DOS of disordered organic semiconductors, such as introduing a modified parameter, a combination of Gaussian DOS and exponential DOS, and so on [14,15,16,17,18,19,20,21]. In other words, most of the current improved DOS models are developed on the basis of Gaussian DOS and exponential DOS. In the final analysis, DOS is a probabilistic description. Therefore, to further reveal the properties of organic semiconductors, combining some appropriate probability theories and statistics, together with physical theories related to DOS, is assumed to obtain a more appropriate functional form of DOS.

In this work, we proposed a novel functional model of DOS based on probability statistics and the frontier orbital theory, instead of an improvement on Gaussian DOS and exponential DOS. It can better fit experimental mobility dependences of charge carriers on both carrier concentration and electric field over a large interval of both variables obtained in organic semiconductors than Gaussian DOS and exponential DOS. In addition, we also provide a detailed method to choose a DOS parameter for better use of the proposed DOS model, and analyze mobility saturation under an electric field. Moreover, a prediction has been made for the DOS parameters and it has been verified by the experimental data. Also, the physical meaning of the proposed DOS parameter is indicated and provides an explanation to the prediction in this paper.

## 2. Model Theory

Generally speaking, the transfer of electrons and holes in disordered organic semiconductors takes place on the LUMO (lowest unoccupied molecular orbital) and the HOMO (highest occupied molecular orbital), respectively, according to the frontier orbital theory [5,22]. In other words, the DOS of the disordered organic semiconductors is a description of the probability distribution of quantum states near the LUMO and HOMO [23,24]. One can obtain the DOS distribution function g(E) by the following equation:

(1) $$g\left(E\right)=\lim_{\Delta E\to 0}\frac{\Delta Z}{\Delta E}=N_{t}g_{1}\left(E\right),$$

here, ΔZ means the number of states between energy *E* and E+ΔE. $N_{t}$ is the number of states per unit volume; it means $\int_{-\infty }^{+\infty }g\left(E\right)dE=N_{t}$. $g_{1}\left(E\right)$ means the probability density of a quantum state at energy *E*.

Most of the $g_{1}\left(E\right)$ probability densities in the previous articles were Gaussian distribution, exponential distribution and their variants [6,7,8,9,10,11,12,14,15,16,17,18,19,20,21]. However, these models are under the premise that all the states in disordered organic semiconductors are localized [7,8], which is inconsistent with Anderson’s localization theory: in a disordered system, states are only localized in the band tail of DOS, and are extended in the center of the band [25]. Moreover, in probability statistics, the Gaussian distribution and exponential distribution describe the distribution of all the samples [26], corresponding to all the molecular orbitals. This contradicts our previous description of DOS which is distributed near the LUMO and HOMO. LUMO (HOMO) are only the outermost energy levels of molecular orbitals that are most likely to gain (lose) electrons, which means they should correspond to the upper bound energy distribution of molecular orbitals, not the energy distribution of all molecular orbitals.

**Figure 1:**
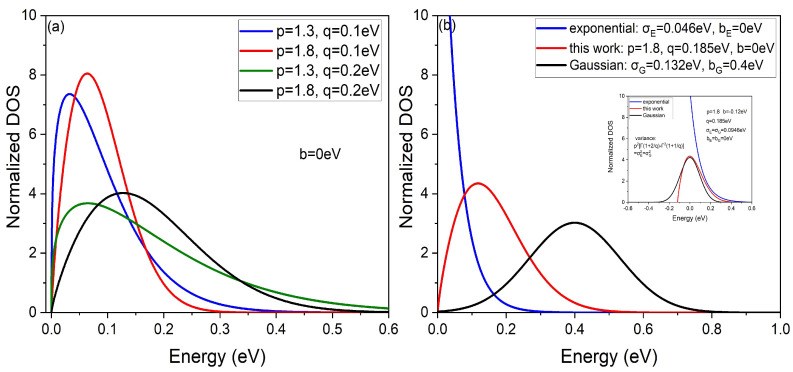
(**a**) The proposed DOS for different parameters *p* and *q*. (**b**) A comparison of different DOS models. Inset: comparison with the same variance.

In order to find the upper bound distribution $g_{1}\left(E\right)$ [27], two problems need to be solved: the number of states is very large, and the energy distribution of all the molecular orbitals is unknown. Fortunately, the extreme value distribution theory in probability statistics, that is the probability distribution of the extreme values of samples given a large sample size, can solve both problems [28]. According to the generalized extreme value theory, no matter what the distribution of total samples (the energy distribution of all the molecular orbitals) is, the extreme value distribution of samples (DOS near the LUMO and HOMO) is satisfied by one of the three distributions of Gumbel, Frechet and Weibull. Since the characteristic functions of the three distributions are essentially consistent [29], the Weibull distribution is selected as $g_{1}\left(E\right)$ and substituted into Equation (1) to obtain the DOS function (See Appendix A for detailed derivation):

(2) $$g\left(E\right)=\left\{\begin{array}{cc} \frac{N_{t}}{p^{-1}q}{\left(\frac{E-b}{q}\right)}^{p-1}\exp\left[-{\left(\frac{E-b}{q}\right)}^{p}\right], & E\geq b\\ 0, & E<b \end{array}\right.$$

here, *p* is the shape parameter and it controls the proportional distribution of the extended and localized states, *q* is the width parameter and it affects the width of DOS more. *b* is the positional parameter and it does not affect the shape, just like $b_{G}$ and $b_{E}$. Figure 1 shows that the proposed DOS should be a positive skewness distribution, neither symmetric, as for Gaussian DOS, nor monotonic, as for exponential DOS.

In addition, *p* can also represent the degree of doping of organic semiconductors to a certain extent. Nowadays, organic semiconductors are more or less doped for higher mobility. As stated by some researchers, with an increase in doping degree of the organic semiconductor, the DOS tail of organic semiconductor will become heavier and the Gaussian peak value will shift. Obviously, as shown in Figure 1a, the smaller *p* is, the heavier the tail of DOS and the greater the Gaussian peak shift extent, that is, the heavier doping of organic semiconductors, which is consistent with the effect of DOS in the previous articles [19,30].

Next, based on Miller–Abrahams (MA) transition rate and the transport energy ($E_{t}$), we modeled to caculate p-type organic semiconductor mobility with concentration, temperature and electric field, to verify the availability of this DOS.

In general, $E_{t}$ can be determined by the equation [31]:

(3) $$\frac{2}{3}{\left(\frac{4\pi }{3B}\right)}^{-\frac{1}{3}}\alpha_{0}k_{B}T{\left[\int_{E_{t}}^{\infty }\left[1-f_{F}\left(E\right)\right]g\left(E\right)dE\right]}^{-\frac{4}{3}}\times \left[1-f_{F}\left(E_{t}\right)\right]g\left(E_{t}\right)=1,$$

here, $\alpha_{0}$ is the inverse localization radius of the MA transition rate, $k_{B}$ is the Boltzmann constant, *T* is the temperature, and *B* is the average number of empty sites in the hopping distance (B≈2.7). $f_{F}\left(E\right)$ is Fermi function ($f_{F}\left(E\right)={\left\{1+\exp\left[\left(E_{f}-E\right)/k_{B}T\right]\right\}}^{-1}$), and $E_{f}$ is the Fermi energy.

Then, based on the MA transition rate, the tunneling distance ($R\left(E_{t}\right)$) can be estimated as [20]:

(4) $$R\left(E_{t}\right)={\left\{\frac{4\pi }{3B}\int_{E_{t}}^{\infty }g\left(E\right)\left[1-f_{F}\left(E\right)\right]dE\right\}}^{-\frac{1}{3}}.$$

Finally, the carrier mobility (μ) with concentration can be determined by the following [6],

(5) $$\mu =\frac{v_{0}q_{e}}{k_{B}T}\frac{3B}{4\pi R\left(E_{t}\right)n}\exp\left(-2\alpha_{0}R\left(E_{t}\right)-\frac{E_{f}-E_{t}}{k_{B}T}\right),$$

Here, $q_{e}$ is the unit charge, $v_{0}$ is the attempt-to-jump frequency of the MA transition rate ($v_{0}\approx 1.0\times 10^{12}s^{-1}$) and *n* is the concentration and its equation is as follows [32]:

(6) $$n=\int_{-\infty }^{+\infty }g\left(E\right)f_{F}\left(E\right)dE$$

It follows that the difference between *b* and $E_{f}$ is determined when *n* is determined. Hence, *b* does not affect the mobility calculation.

Furthermore, the effective drift hopping mobility ($\mu_{e}$) under the electric field can be obtained as [9]:

(7) $$\mu_{e}=d\times \left(1-\frac{\int_{-\infty }^{+\infty }g\left(E\right)f_{F}^{2}\left(E\right)dE}{\int_{-\infty }^{+\infty }g\left(E\right)f_{F}\left(E\right)dE}\right)\times \frac{W_{e}^{+}-W_{e}^{-}}{F},$$

here, $d=N_{t}^{-\frac{1}{3}}$, *F* is electric field, $W_{e}^{+}$ and $W_{e}^{-}$ are the effective jump rates, and they can be calculated by [16]:

(8) $$W_{e}^{\pm }=\frac{\int_{E_{t}}^{+\infty }g\left(E\right)\left[1-f_{F}\left(E\right)\right]dE}{\int_{E_{t}}^{+\infty }g\left(E\right)\left[1-f_{F}\left(E\right)\right]{\left\{W_{12}^{\pm }\left(E_{t},E\right)\right\}}^{-1}dE},$$

Here, $W_{12}^{+}$ and $W_{12}^{-}$ are effective jump rates between two neighboring localized sites along and opposite to the electric field direction, respectively, which can be calculated by the MA transition rate below [21,33]:

(9) $$W_{12}^{\pm }\left(E_{t},E\right)=v_{0}\exp\left(-2\alpha_{0}R\left(E_{t}\right)\right)\exp\left[-\frac{\left|E-E_{t}\mp q_{e}dF\right|+\left(E-E_{t}\mp q_{e}dF\right)}{2k_{B}T}\right].$$

## 3. Results and Discussion

**Figure 2:**
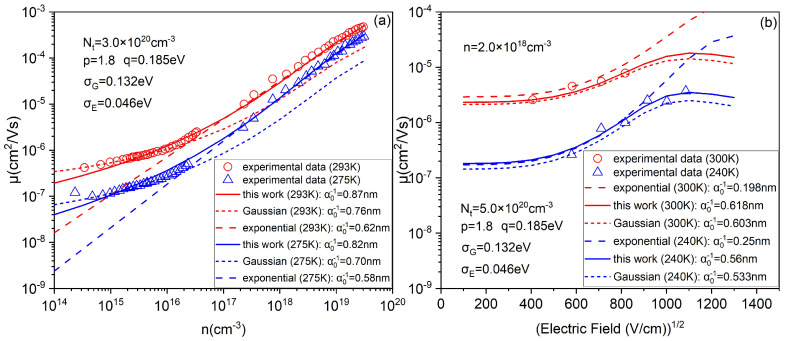
(**a**) A comparison between the different DOS and experimental data (Ref. [34]) of the concentration dependence of the mobility. (**b**) A comparison between the different DOS and experimental data (Ref. [10]) of the electric field dependence of the mobility.

First, we verified the feasibility of the proposed DOS by comparing the different DOS simulation results with the experimental data on the temperature, carrier concentration and electric field dependence of the mobility [10,34]. Figure 2a shows that the model based on exponential DOS deviates from the experimental data at low concentrations, as well as that based on the Gaussian DOS at high concentrations. The fundamental reason is that the proportion of extended states and localized states is inconsistent with reality. At low concentration, carriers are mainly concentrated in the tail of the DOS in the form of localized states. However, according to the superposition state of the wave function (the exponential form of the one-electron approximation) and the univariate description of exponential distribution in statistics, exponential DOS treats all states as extended states, resulting in large variations in mobility even at low concentrations. On the contrary, for the carrier distribution at high concentrations, other than the tail of DOS (localized states), the middle part of DOS (extended states) should also be considered. However, the premise of Gaussian DOS is treating all states as localized states, which results in the slow growth of mobility even at high concentrations. By contrast, at the same variance and peak energy as shown in Figure 1b, the proposed DOS has better convergence in the tail (localized states) than the exponential DOS. On the other hand, compared with the symmetric Gaussian DOS, the positive bias characteristic of the proposed DOS has more extended state proportion (high concentration), which is consistent with the exponential DOS. That is why the proposed DOS is effective over a wide range of concentrations. Figure 2b shows, compared with other models, that the model based on the proposed DOS also can better fit the experimental data, especially under high electric field. It is because Gaussian DOS has more localized states than the reality, resulting that the mobility is lower than the experimental data, when the electric field provides more activation energy. Similarly, the situation is opposite for exponential DOS (more extended states).

Then, we explain the rationality of this proposed DOS and the physical meaning of its parameters by equilibrium energy theory ($E_{\infty }$) and transport energy ($E_{t}$) theory [35]. It is well known that the carrier energy of disordered organic semiconductors at low concentrations does not decline with time indefinitely, but stays around a certain energy in most of the time, which is also called equilibrium energy theory ($E_{\infty }$) [36]. It can be determined by the following equation [35,36]:

(10) $$E_{\infty }=\frac{\int_{-\infty }^{\infty }Eg\left(E\right)f_{F}\left(E\right)dE}{\int_{-\infty }^{\infty }g\left(E\right)f_{F}\left(E\right)dE}\approx \frac{\int_{-\infty }^{\infty }Eg\left(E\right)\exp\left(E/k_{B}T\right)dE}{\int_{-\infty }^{\infty }g\left(E\right)\exp\left(E/k_{B}T\right)dE}$$

According to this equilibrium energy theory, we can know that when Fermi energy $E_{f}\left(n\right)>E_{\infty }$, the mobility is a definite value which does not change with the concentration. When Fermi energy $E_{f}\left(n\right)\leq E_{\infty }$, the mobility will increase rapidly with the concentration [6]. Therefore, we can determine the critical concentration value ($n_{c}$), above which the mobility will change from a definite value to a rapid change value with the concentration, by the following equation:

(11) $$E_{f}\left(n_{c}\right)\approx E_{\infty }$$

Obviously, one can obtain $n_{c}$ easily, by substituting Equations (6) and (10) into Equation (11). On the other hand, similar to the common multiple-trapping model (MTR) with the mobility edge, energy relaxation of carriers is replaced by some particular energy, which is called transport energy ($E_{t}$) [37,38]. Moreover, the relaxation time ($t_{r}$) that it takes for carriers to get from $E_{\infty }$ to $E_{t}$ by thermal activation, can be estimated by [36]:

(12) $$t_{r}\approx v_{0}^{-1}\exp\left(E_{\infty }/k_{B}T\right)\exp\left(2\alpha_{0}N_{t}^{-\frac{1}{3}}\right)$$

**Figure 3:**
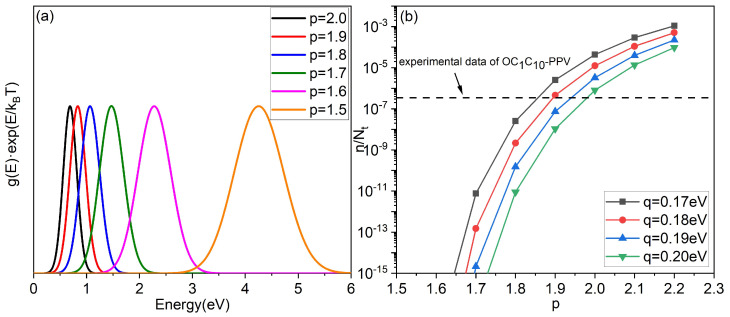
(**a**) $g\left(E\right)\exp\left(E/k_{B}T\right)$ as a function of energy for different *p* and q=0.185eV. (**b**) the critical concentration $n_{c}$ as a function of the proposed DOS parameter *p* for different parameter *q*. The dashed lines represent the experimental data with the lowest concentrations in Figure 2a.

Then, based on the above theory, we can predict the reasonable interval of *p*. The curve of $g\left(E\right)\exp\left(E/k_{B}T\right)$ with different *p* is shown in Figure 3a. According to the properties of the integral and Equation (10), it is obvious that the energy value at the peak of the curve corresponds to $E_{\infty }$. At the same time, the maximum energy of DOS is approximately equal to $E_{t}$. The calculation by Equation (12) shows that when p=1.6, $t_{r}$ is greater than $\nu_{0}^{-1}\exp\left(92\right)$. This is obviously much larger than the realistic experimental situation [6]. Hence, the value of *p* should be higher than 1.6.

**Figure 4:**
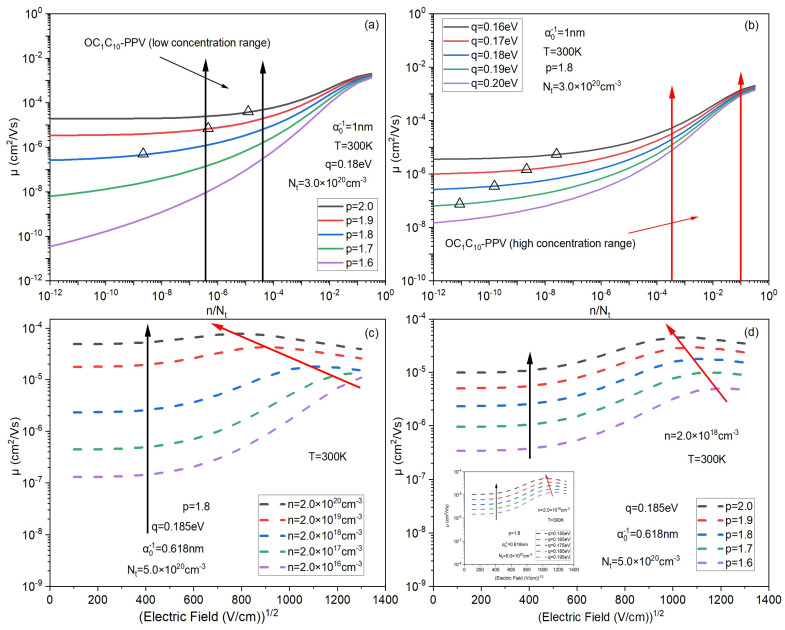
(**a**,**b**) The effect of parameter *p* and *q* on mobility with concentration. The triangle in (**a**,**b**) represents the point of $n_{c}$ calculated in Figure 3b. (**c**) Effect of concentration on mobility with electric field. (**d**) Effect of DOS parameters on mobility with electric field. The black arrow in (**c**,**d**) represents the location where rapid growth of mobility begins, and the red arrow in (**c**,**d**) represents the location of saturation.

Moreover, according to Equations (6), (10) and (11), the relationship between the critical concentration $n_{c}$ and *p* for different *q* is shown in the Figure 3b. The fitting parameter $N_{t}=3.0\times 10^{20}\;{\mathrm{cm}}^{-3}$, just like Figure 2a. It is clear from Figure 2a that for $OC_{1}C_{10}-\mathrm{PPV}$, from the experimental data point at the lowest concentration, mobility has begun to increase with concentration. This means that the value of $n_{c}$ should be less than the concentration value at its lowest experimental data point (below the dashed line in Figure 3b). Obviously, for $OC_{1}C_{10}-\mathrm{PPV}$, the value of $n_{c}$ is in the scope which is below the dashed line only when p<1.9. In addition, we labeled the data of Figure 3b in Figure 4a,b to verify the validity of the $n_{c}$ values (q=0.18eV and p=1.8). Through these data points (marked in triangle), it can be seen that the standard of $n_{c}$ is basically met. When the concentration is greater than these data points, the mobility begins to increase with an increase in the concentration, which also indicates the rationality of adopting this equilibrium energy model. According to the above analysis, 1.7<p<1.9 is more reasonable for $OC_{1}C_{10}-\mathrm{PPV}$.

**Figure 5:**
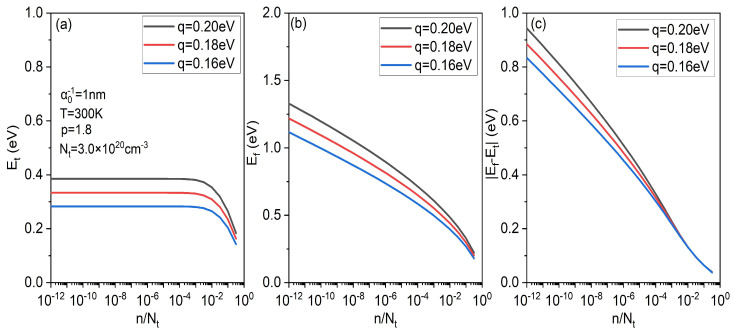
(**a**) $E_{t}$ as a function of concentration *n* for different *q*. (**b**) $E_{f}$ as a function of concentration *n* for different *q*. (**c**) the difference between $E_{t}$ and $E_{f}$ as a function of concentration *n* for different *q*.

Then, we make a simple analysis of width parameter *q* through the relationship between Fermi energy $E_{f}$, transport energy $E_{t}$ and concentration *n* under different *q*, as shown in Figure 5. Figure 5a shows that at low concentration, the transport energy is basically unchanged no matter what the value of *q* is. It is well known that charges below the transport level do not contribute to the conduction of electricity. This is the reason why the mobility is basically unchanged at low concentration. On the other hand, from Figure 5b,c, with an increase in concentration, the Fermi level drops faster and faster, which makes the difference between the transport energy and Fermi energy smaller and smaller. That is one of the reasons for the rapid increase in mobility with concentration at high concentration. Moreover, at high concentration, the greater the *q*, the greater the slope of the difference between $E_{t}$ and $E_{f}$ with concentration, that is, the greater the *q*, the greater the increase in mobility with concentration, as shown in Figure 4b.

Next, we calculated the influence of parameters *p* and *q* on mobility to further verify the rationality of this DOS. As shown in Figure 2a, the mobility of OCC-PPV increases by only about a factor of 5 in the low concentration interval, but nearly 100 times in the high concentration interval. The larger parameter *p*, the greater the proportion of localized states, and thus the slower the mobility grows at low concentration. Hence, the value of *p* can be determined according to the growth degree of mobility in the low concentration interval. Figure 4a shows that the growth degree of mobility at p=1.8 is closest to the growth degree of the experimental data in Figure 2a (about 5 times). More importantly, this is consistent with our previous predictions. Then, the larger parameter *q*, the larger DOS width, the greater change in Fermi level as the concentration changes, results in the greater change in activation energy and the greater change in mobility at high concentrations, which is also consistent with our previous analysis of Figure 5. Therefore, after determining *p*, the value of *q* can be determined according to the increasing degree of mobility in the high concentration interval, as shown in Figure 4b. Then, the proposed DOS comes out.

In addition, we also analyzed the mobility saturation under the electric field. Figure 4c shows that when $\sqrt{F}>400{\left(\;V/\mathrm{cm}\right)}^{0.5}$, mobility begins to increase rapidly with the enhancement of the electric field, conforming to the Poole–Frenkel rule, until saturation is reached [39]. The lower the concentration, the higher the electric field reaching mobility saturation is. It is because the localized state is dominant at low concentration which causes more activation energy (higher electric field) to be needed to saturate the mobility. Figure 4d shows that when different DOS are at the same concentration, the smaller *p* is, the greater the growth degree of mobility with the electric field increasing and the greater the saturation field strength. That is because the smaller *p* is, the closer the proposed DOS is exponential DOS, as shown in Figure 2b. Obviously, Figure 4d shows that the influence on mobility with electric field of shape parameter *p* is greater than width parameter *q*.

## 4. Conclusions

In summary, we proposed a novel DOS for the disordered organic semiconductors. The proposed DOS exhibits a positive skewness distribution, neither symmetric as Gaussian DOS nor monotonic as exponential DOS. The mobility model based on the proposed DOS can well fit the experimental data at both high and low concentrations, as well as the electric field dependent on the mobility. Furthermore, we used equilibrium energy theory and transport energy theory to explain the physical meaning of the proposed DOS parameters and make prediction. Through calculation and analysis, the final results are consistent with the experimental data and our prediction is verified. In addition, the good fitting effect of the mobility based on this DOS is also a kind of testimony of Anderson’s localization theory. This proposed DOS provides impressive potential for future investigation on charge carrier transport in organic semiconductors.

## Appendix A

Physically, the DOS is the number of quantum states (ΔZ) with energies between *E* and E+ΔE, i.e., the ratio of ΔZ to ΔE. According to the definition of DOS, one can obtain the following equation:

(A1) $$g\left(E\right)=\lim_{\Delta E\to 0}\frac{\Delta Z}{\Delta E}=Ng_{1}\left(E\right),$$

here, *N* is the number of states per unit volume. It means ∫g(E)dE=N. $g_{1}\left(E\right)$ means the probability density of a quantum state at energy *E*.

Firstly, one can use the random variable $E_{k}$ (such as $E_{1},E_{2},\ldots ,E_{k},\ldots ,E_{N-1},E_{N}$) to represent the energy of the localized state of every molecular orbital in organic semiconductors, respectively. It should be noted that $E_{k}$ is a variable, not a constant. As is well known, the state of a single particle is described by a wave function, which is a probability wave [40]. As the electron cloud shows, the tiny black spot on the electron cloud just represents the probability of electron appearing, instead of the electron actually existing [41]. The same situation applies to the energy of states. That is, a particular energy value of states at high probability does not represent the particular value is the actual value. Otherwise, if one assumes that each state is localized, then the position of the state is approximately fixed, and the energy of state is certain. That will cause a contradiction with the uncertainty principle [42].

Therefore, in order to describe the random variables of $E_{k}\left(1\leq k\leq N,k\in Z\right)$ more accurately, one can assume that the distribution of the random variables of $E_{k}$ is function f(E), which means the probability density is f(E) when $E_{k}=E$. Then, the probability F(E) can be defined as,

(A2) $$F\left(E\right)=\int_{-\infty }^{E}f\left(t\right)dt,$$

where F(E) is the probability at which the energy is lower than *E*. Moreover, f(E) is related to the material composition of the organic semiconductor.

Next, we will discuss the forming process of DOS in the disordered system. In the molecule, the DOS of the p-type disordered organic semiconductor is the number distribution of the states at various energy levels near the HOMO, such as HOMO, HOMO-1, HOMO-2 and so on [5]. The HOMO is filled with the highest energy electrons, and it is the place where molecules are most likely to lose electrons, similar to the valence band of the inorganic semiconductor. According to Anderson’s localization theory and Mott’s theory of mobility edges, in a disordered system, electrons are localized in the band tail of DOS, and are extended in the center of the band. The regions of these two kinds of states are divided by mobility edges [25]. In addition, the greater the disorder, the narrower the region of extended states and the wider the region of tail states. In the published articles, researchers always assumed that molecular orbitals are all in localized states, and then supposed a DOS distribution function to calculate the carrier transport under different conditions, and verified the feasibility through the comparison between experimental results and simulation results [7,8,14,18]. To fit the charge transport well, DOS vary in different situations, lacking the theoretical explanation. The fundamental reason is that the distribution of DOS is a macroscopic approximation to the theory of Anderson and Mott, which is not accurate enough. If all states are treated as localized states, the existence of extended states in band center needs to be dealt correctly. In other words, if DOS is considered on HOMO in disordered organic semiconductors, and it should be the distribution of the upper bound of the function f(E), not the distribution of the whole orbitals which is f(E). In this work, one can solve the above problem by applying the following definition at the beginning:

(A3) $$E^{*}:=\sup\left\{E:F\left(E\right)<1\right\},$$

here, sup{} means the upper bound of a set. Physically, it represents the state of each molecular orbital on the HOMO. To facilitate understanding, we take the Gaussian distribution as an example. In general, if one takes enough samples, the sampling distribution will be Gaussian distribution, according to the central limit theorem in statistics. Then, the Gaussian distribution corresponds to the distribution of the entire molecular orbital, not the HOMO, for the reason that the HOMO means highest occupied molecular orbital, which corresponds to the upper bound of the Gaussian.

Then, we will analyze and derive DOS function in the disordered system. To calculate $E^{*}$, one can adopt the maximum of random variables $E_{k}\left(1\leq k\leq N,k\in Z\right)$ to approach $E^{*}$:

(A4) $$\max\left(E_{1},E_{2},\ldots E_{k},\ldots E_{N-1},E_{N}\right)\to E^{*},\;N\to \infty$$

here, max() means maximum value in a set. At a moment, we make $E_{1},E_{2},\ldots ,E_{N-1},E_{N}$ from variable to certain, then the maximum of them can be obtained. Applying this action to different moments, we can obtain infinite numbers of maxima, which approach $E^{*}$.

To prove Equation (A4), one can set δ to be any value greater than 0. Then, one can obtain:

(A5) $$\begin{array}{cc} \; & P\left(\left|\max\left(E_{1},\ldots E_{k},\ldots E_{N}\right)-E^{*}\right|>\delta \right)=P\left(\max\left(E_{1},\ldots E_{k},\ldots E_{N}\right)<E^{*}-\delta \right)\\ \; & =P\left(E_{1}<E^{*}-\delta ,\ldots ,E_{N}<E^{*}-\delta \right)=F\left(E^{*}-\delta \right)\times \ldots \times F\left(E^{*}-\delta \right)\\ \; & =F^{N}\left(E^{*}-\delta \right)\to 0,\;N\to \infty \end{array}$$

Here, P() means the probability of an event occurring in parentheses. Obviously, $F\left(E^{*}-\delta \right)<1$, and the number *N* of states per unit volume is approaching to infinity. Then, Equation (A4) is proved.

Then, according to the definition of Equations (A3) and (A4), one can obtain the following equation:

(A6) $$\begin{array}{cc} \; & \int_{-\infty }^{E}g_{1}\left(t\right)dt=P\left(E^{*}<E\right)=P\left(\max\left\{E_{1},E_{2},\ldots ,E_{k},\ldots ,E_{N}\right\}<E\right)\\ \; & =P\left(E_{1}<E,E_{2}<E,\ldots ,E_{k}<E,\ldots \right)=\prod_{i=1}^{N}P\left(E_{i}<E\right)=F^{N}\left(E\right), \end{array}$$

Here, $\int_{-\infty }^{E}g_{1}\left(t\right)dt$ corresponds to the probability that a quantum state is lower than *E*.

Finally, substituting Equation (A6) into Equation (A1), the equation of DOS in the disordered system can be written as

(A7) $$g\left(E\right)=Ng_{1}\left(E\right)=N\frac{d\int_{-\infty }^{E}g_{1}\left(t\right)dt}{dE}=N\frac{dF^{N}\left(E\right)}{dE}.\;$$

For more universality of this model, f(E) and F(E) will be set to arbitrarily reasonable distributions, and *N* tends to infinity. Based on Fisher and Tippet theory [28], one can obtain the following equation:

(A8) $$F^{N}\left(E\right)=\exp\left\{-{\left(1+\gamma \frac{E-b}{a}\right)}^{-\frac{1}{\gamma }}\right\}\;N\to \infty ,1+\gamma \frac{E-b}{a}>0$$

Here, *a* is the width parameter, *b* is the positional parameter, and γ is the shape parameter. When γ is positive, 0, and negative, it corresponds to Frechet distribution, Gumbel distribution and Weibull distribution, respectively.

Then, in terms of Jenkinson theory [29], the positive and negative properties of γ do not affect its eigenfunctions and functional properties. Hence, we could choose Weibull distribution as $g_{1}\left(E\right)$ for ease of calculation.
